# DNA methylation signature of long noncoding RNA genes during human pre-implantation embryonic development

**DOI:** 10.18632/oncotarget.18072

**Published:** 2017-05-23

**Authors:** Jingyu Li, Wei Han, Xiaoli Shen, Shubiao Han, Hong Ye, Guoning Huang

**Affiliations:** ^1^ Chongqing Reproductive and Genetics Institute, Chongqing 400013, China

**Keywords:** long noncoding RNA (lncRNA), pre-implantation embryonic development, DNA methylation, reduced representation bisulphite sequencing

## Abstract

DNA methylation have crucial roles in regulating the expression of developmental genes during mammalian pre-implantation embryonic development (PED). However, the DNA methylation dynamic pattern of long noncoding RNA (lncRNA) genes, one type of epigenetic regulators, in human PED have not yet been demonstrated. Here, we performed a comprehensive analysis of lncRNA genes in human PED based on public reduced representation bisulphite sequencing (RRBS) data. We observed that both lncRNA and protein-coding genes complete the major demethylation wave at the 2-cell stage, whereas the promoters of lncRNA genes show higher methylation level than protein-coding genes during PED. Similar methylation distribution was observed across the transcription start sites (TSS) of lncRNA and protein-coding genes, contrary to previous observations in tissues. Besides, not only the gamete-specific differentially methylated regions (G-DMRs) but also the embryonic developmental-specific DMRs (D-DMRs) showed more paternal bias, especially in promoter regions in lncRNA genes. Moreover, coding-non-coding gene co-expression network analysis of genes containing D-DMRs suggested that lncRNA genes involved in PED are associated with gene expression regulation through several means, such as mRNA splicing, translational regulation and mRNA catabolic. This firstly provides study provides the methylation profiles of lncRNA genes in human PED and improves the understanding of lncRNA genes involvement in human PED.

## INTRODUCTION

Long noncoding RNA (lncRNA) are a class of transcripts that are longer than 200 nucleotides without protein coding capacity. LncRNA genes can be classified into intergenic and intragenic, according to their genome localization. Though the molecular basis of the function of many lncRNA genes is just emerging, the recent work indicates their intricate roles in various biological processes, such as X chromosome inactivation [1], imprinting [2], Hox-associated pattern formation [3, 4], neuronal fate specification [5], pluripotency and differentiation control [6–8], cell apoptosis and cell cycle control [9, 10], immune response [11, 12], and mitochondria regulation [13]. LncRNA genes share many characteristics of protein-coding genes. For instance, most lncRNA genes are transcribed by RNA pol II and have typical hallmarks of pol II transcribed products like 5′ Cap and poly A tail [14]. Therefore, the expression of both lncRNA and protein-coding genes mediated by pol II can be regulated by DNA methylation alterations [15].

In recent years, several studies have implicated lncRNA genes in mammalian pre-implantation embryonic development (PED). Recently, one study reports a bidirectional promoter-associated lncRNA named *pancIl17d* playing key roles in mouse PED [16]. In a different study, we identified a novel endogenous retroviruses associated lncRNA, *lncGET*, which is essential for mouse PED beyond the two-cell stage via regulating the transcription and RNA alternative splicing at major zygotic genome activation (ZGA) stage [17]. However, because the sequence conservation of lncRNA is very low, the extrapolation to human PED is limited.

It is well known that DNA methylation is a key regulator of gene expression [18]. DNA methylation is highly dynamic and changes extensively during mammalian PED [19–23]. Accurate dramatic changes of methylome, including demethylation after fertilization and re-methylation during implantation, are essential for the successful PED. Considering that lncRNA genes can regulate gene expression via various mechanisms at the pre-transcriptional [3, 4, 24–28], transcriptional [29], and post-transcriptional levels [30, 31]. The expression alterations of lncRNA genes mediated by methylation alterations can subsequently affect their downstream genes. However, unlike the protein-coding genes, a systematic analysis of DNA methylation features in lncRNA genes during PED has not yet been undertaken.

In this study we performed genome-wide analysis of the DNA methylation of lncRNA genes in human pre-implantation embryos. Comparison of the methylation patterns observed in lncRNA and protein-coding genes identified several distinctive methylation characteristics that differ between these classes of genes. We also analyzed gamete-specific DMRs (G-DMRs) and developmental-specific DMRs (D-DMRs) in lncRNA genes. To investigate the potential roles of lncRNA genes related to the promoter D-DMRs, we performed coding–non-coding gene co-expression (CNC) network analysis, and revealed they display strong association with gene expression regulation. We believe our comprehensive methylation analysis of lncRNA genes would help on a better understanding of molecular regulations that occur in human PED.

## RESULTS

### Global pattern of DNA methylation in lncRNA genes during human PED

**Figure 1:**
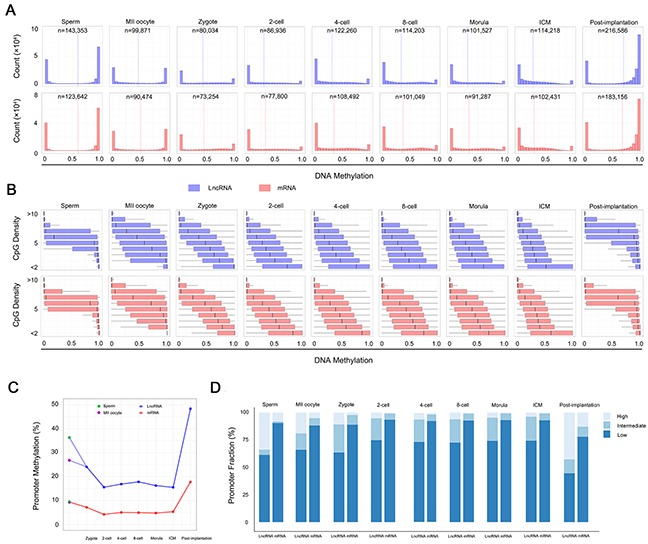
DNA methylation features of lncRNA genes during human PED **(A)** DNA methylation of lncRNA and protein-coding genes across 100-bp tiles for each developmental stage. Dashed line highlights the average. n equals the total number of the 100-bp tiles for a given stage. **(B)** Boxplots of methylation at different local CpG densities (*y* axis). **(C)** Promoter methylation of lncRNA and protein-coding genes across each developmental stage. Blue, lncRNA genes. Red, protein-coding genes. **(D)** Histogram of promoter fractions of 100-bp tiles with three different methylation status across different developmental stages. The tiles were divided into three status based on the methylation level: high (> 80%), intermediate (20% - 80%) and low (< 20%).

Previous studies have shown that lncRNA genes have several characteristics which differ from those of protein-coding genes such as length, number of exons and level of expression [32, 33]. Here, we found that lncRNA and protein-coding genes share similar architectures in DNA methylation during human PED: a strong CpG-density-dependent bimodality in sperm and post-implantation embryos, while is intermediate in oocyte and weaker in pre-implantation embryos (Figure 1A, 1B). When we analyzed the genomic regions separately, such as the promoter, intron and exon regions, the dynamic patterns of lncRNA genes of demethylation and re-methylation were similar to those of protein-coding genes, indicating that the dynamic changes in DNA methylation are in general universal in the two types of genes during human PED (Figure 1C and Supplementary Figure 1). Interestingly, we found that the methylation levels in promoter of the lncRNA genes were always higher than that of protein-coding genes during human PED (Figure 1C, 1D and Supplementary Figure 2), suggesting that there exists differential methylation pattern between lncRNA and protein-coding genes. The results may reflect differential methylation regulation mechanisms between lncRNA and protein-coding genes.

### Distribution of DNA methylation across TSS of lncRNA genes

**Figure 2:**
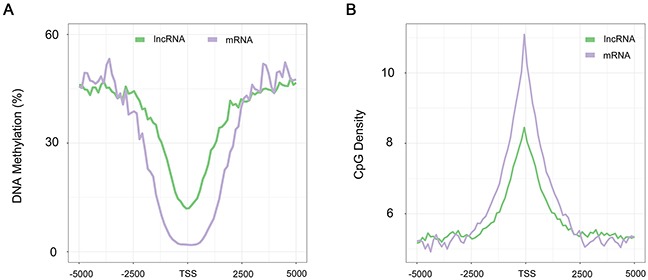
DNA methylation patterns around the TSS of lncRNA genes **(A)** Distribution of the methylation level calculated in 100-bp sliding windows, 5-kb up- and downstream from the TSS in sperm. **(B)** Distribution of CpG densities across the TSS of lncRNA and protein-coding genes. Green, lncRNA. Purple, protein-coding genes.

The methylation architecture in and around the protein-coding genes is important for gene expression and cell identity [18]. In an earlier study, sati *et al*. found the methylation density around TSS was markedly different between lncRNA and protein-coding genes in human H1 cell line and brain tissue, with a V-shaped curve in protein-coding genes and a sharp peak immediately downstream of the transcription start site (TSS) in lncRNA genes [34]. However, our results showed similar methylation distribution in lncRNA and protein-coding genes during human PED, with a V-shaped curve indicative of a relative low methylation level around the TSS (Figure 2A). Furthermore, we found that the average methylation density around TSS of lncRNA genes was in overall higher than that of protein-coding genes during human PED (Figure 2A and Supplementary Figure 3; Kolomogorv-Smirnov Test, *P*-value < 2.2 × 10^−16^). The different observation with the previous study may be due to the special cell stage, pre-implantation embryo, where carrying out the most dramatic genome-wide changes of the methylome. To rule out the influence due to lncRNA genes that fall within protein-coding genes, only lncRNA gene that lie at least 1 kb away from a protein-coding gene were analyzed.

Several studies have shown that there is a strong correlation between CpG density and transcription initiation [35]. We thus plotted the CpG density across the TSS of lncRNA genes to assess if the promoters of lncRNA genes are also rich in CpG, and found that it is true for lncRNA genes but the CpG density was considerably lower compared to protein-coding genes (Figure 2B; Kolomogorv-Smirnov Test, *P*-value < 2.2 × 10^−16^).

### Relationships between DNA methylation and lncRNA gene expression

**Figure 3:**
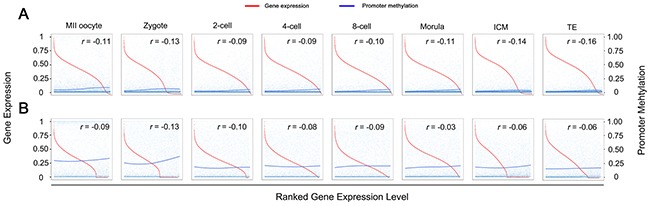
Relationships between DNA methylation level of promoter and gene expression during human PED **(A)** The scatter plot of DNA methylation levels of promoter regions and the relative expression levels of corresponding protein-coding genes. The log_2_ of the gene expression levels (FPKM) were calculated and are presented. The Pearson correlation coefficients (*r*) between DNA methylation levels of promoter regions and the scaled expression levels of the corresponding genes across every developmental stage were calculated and are included in the top right corner of each panel. The red and blue fitting curves in each display represent gene expression levels and DNA methylation levels in promoter regions, respectively. The horizontal axis from left to right below each box represents the expression levels from high to low of protein-coding genes, respectively. **(B)** The same in **(A)** of lncRNA genes.

We incorporated recently published single-cell transcriptome data to investigate the relationship between DNA methylation and lncRNA genes expression [36]. As expected, both lncRNA and protein-coding genes expression negatively correlate with promoter methylation across human PED (Figure 3A, 3B). Interestingly, despite more dramatic changes in promoter methylation of lncRNA genes occurred during PED than that of protein-coding genes (Figure 1C), the promoter methylation of lncRNA genes displayed lower strength on expression repression, especially after ZGA at the 8-cell stage (Figure 3A, 3B). These results suggested that lncRNA genes might be subjected to DNA methylation regulation resembling protein-coding genes, and further indicated the differential regulation of DNA methylation between lncRNA and protein-coding genes. Notably, the DNA methylation on the gene bodies showed positive correlation with the expression levels of corresponding lncRNA and protein-coding genes during human PED (Supplementary Figure 4A, 4B).

### Gamete-specific DMRs located in lncRNA and protein-coding genes

**Figure 4:**
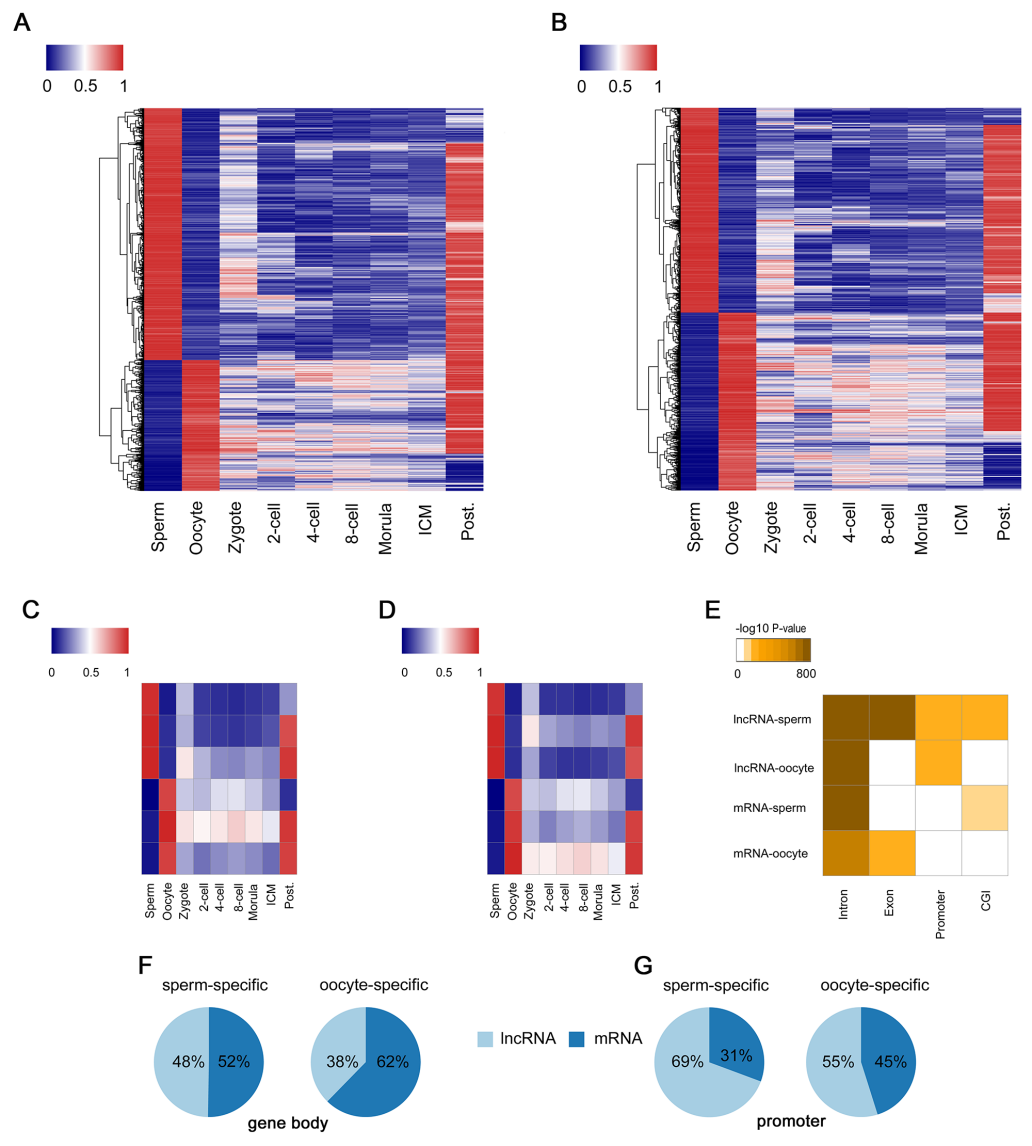
Identification of G-DMRs in lncRNA and protein-coding genes **(A)** Heat map of the methylation level of G-DMRs in lncRNA across each developmental stages. **(B)** The same in **(A)** of protein-coding genes. **(C)** G-DMRs in lncRNA regions are clustered via k-means into 6 dynamics. **(D)** The same in **(C)** of protein-coding genes. **(E)** The hypergeometric enrichment analysis of different G-DMRs including sperm-specific DMRs in lncRNA (lncRNA-sperm), lncRNA-oocyte, mRNA-sperm and mRNA-oocyte for intronic, exonic, promoter or CGI annotations, which indicates the different methylation features of G-DMRs between lncRNA and protein-coding genes. **(F)** The ratio of lncRNA and protein-coding genes in sperm-specific or oocyte-specific DMRs among gene body regions. **(G)** The ratio of lncRNA and protein-coding genes in sperm-specific or oocyte-specific DMR among promoter regions.

DMRs contributed by the two gametes, including known imprint control regions (ICRs), play indispensable roles during human PED [37]. We thus systematically searched for G-DMRs in lncRNA and protein-coding genes (Figure 4A, 4B). Notably, we found that the G-DMRs in lncRNA genes were more paternal bias compared to protein-coding genes (67% and 51%; Chi-square Test, *P*-value < 2.2×10^−16^). We clustered the G-DMRs using k-means into 6 dynamic patterns separately, and found similar DNA methylation dynamics between lncRNA and protein-coding genes (Figure 4C, 4D). Sperm-specific DMRs in both types of genes rapidly lose methylation before the 2-cell stage and retain only background levels of methylation. On the contrary, many of the oocyte-specific DMRs in both lncRNA and protein-coding genes displayed imprint-like DNA methylation patterns during PED with an average methylation level around 50%. Moreover, the majority of G-DMRs in both lncRNA and protein-coding genes were remethylated during implantation. Interestingly, we found that the sperm-specific DMRs in lncRNA genes were enriched in exon regions, which was similar to the oocyte-specific DMRs of protein-coding genes (Figure 4E). In addition, G-DMRs overlapping with promoter regions localize to lncRNA genes more frequently than protein-coding genes (Figure 4E, 4F, 4G). Therefore, we postulated that the different methylation patterns between lncRNA and protein-coding genes contribute importantly to the parent-of-origin methylation maintained during human PED.

### Identification and functional analysis of D-DMRs located in lncRNA genes

**Figure 5:**
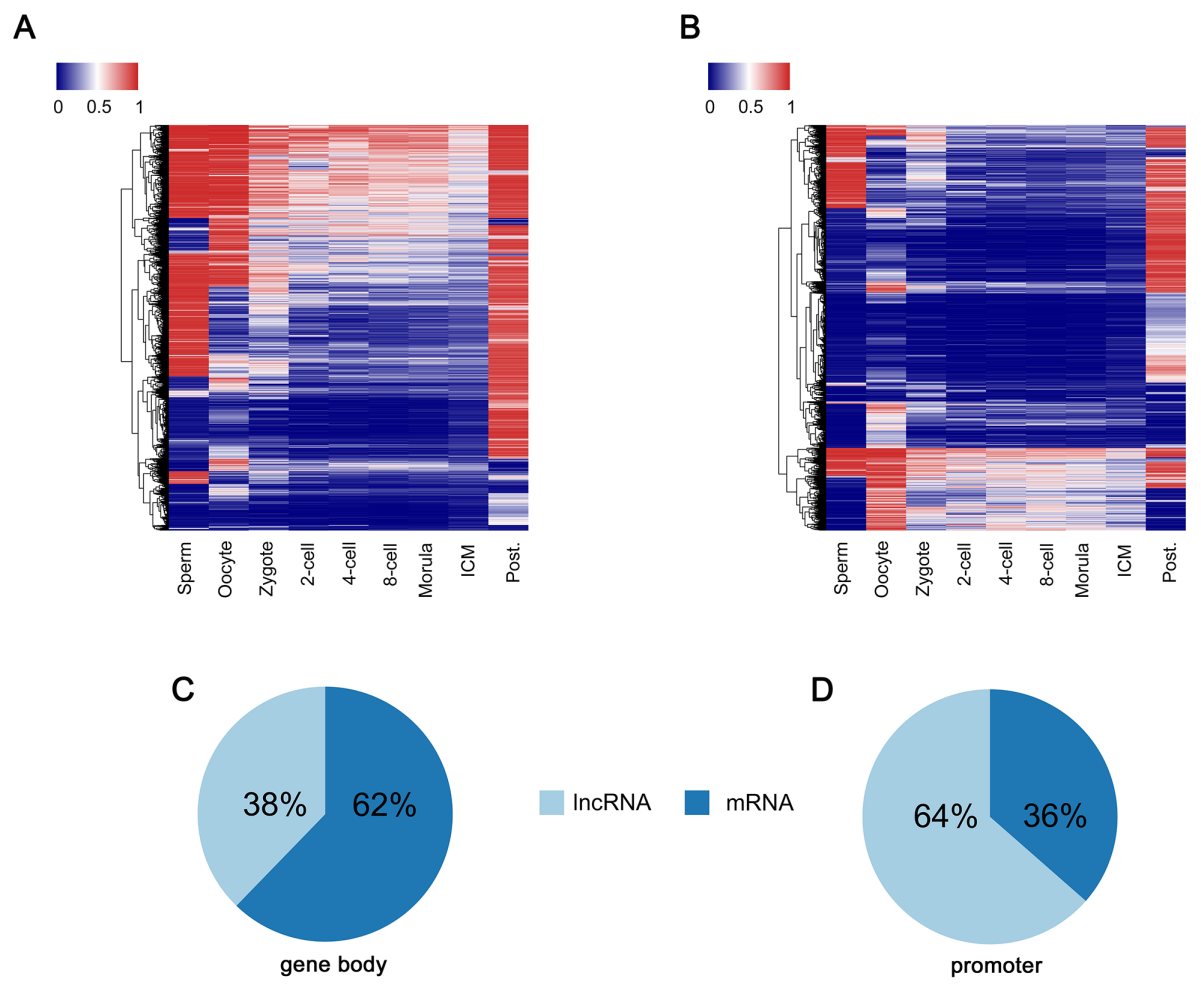
D-DMRs analysis through QDMR based on entropy **(A)** Heat map of the methylation level of D-DMRs in lncRNA regions across each developmental stages. **(B)** Heat map of the methylation level of D-DMRs in protein-coding gene regions across each developmental stages. **(C)** The ratio of lncRNA and protein-coding genes in D-DMRs among gene body regions. **(D)** The ratio of lncRNA and protein-coding genes in D-DMRs among promoter regions.

DMRs among multiple samples (tissues, cells or others), are regarded as potential functional regions involved in gene transcriptional regulation [38]. In this study, we applied quantitative differentially methylated regions (QDMR) method [39] to quantify methylation difference across human PED, and identified a total of 82,066 D-DMRs according to the threshold HDMR=4.22 (Supplementary Dataset 1). The heat map demonstrated that D-DMRs in lncRNA genes were also more paternal bias compared to protein-coding genes (71% and 22%; Chi-square Test, *P*-value < 2.2×10^−16^; Figure 5A, 5B). In addition, the radio of D-DMRs overlapping with the promoter of lncRNA genes was about 2-fold as that with protein-coding genes (64% versus 36%; Chi-square Test, *P*-value < 2.2×10^−16^; Figure 5D). These results were consistent with our findings in G-DMRs analysis.

**Figure 6:**
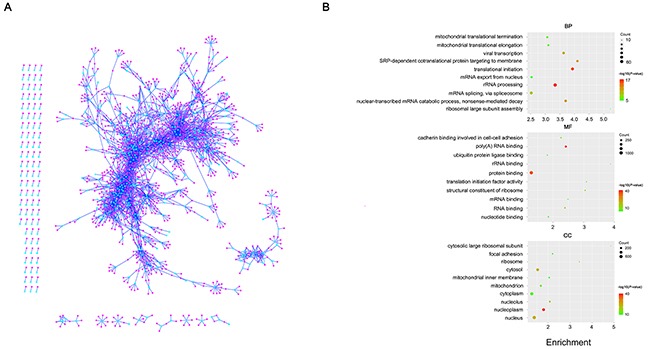
The coding-non-coding gene co-expression network **(A)** Visualization of the CNC network. Cyan nodes represent lncRNA genes while pink nodes represent protein-coding genes. **(B)** Function enrichment analysis result of protein-coding genes in the CNC network. The colors indicate the significance (-log10 transferred *P*-value), and the circle size represent the number of genes enriching the corresponding annotation. The fold enrichment of analysis are shown in horizontal axis. Top, “biological process” term. Middle, “molecular function” term. Bottom, “cellular component” term.

Previous studies found that promoter D-DMRs are associated with genes that are thought to function in a specific manner [40]. Here, we analyzed the functions of the lncRNA genes related to the promoter D-DMRs identified by QDMR in human PED. Becacuse the exact functions of the majority of lncRNA genes are still unknown, we performed CNC network analysis to investigate the potential roles of lncRNA genes related to the promoter D-DMRs in human PED. To this end, transcriptome data sets were used to construct CNC network including the lncRNA genes related to promoter D-DMRs. In our CNC network, there were 509 lncRNA and 1,812 protein-coding genes that were linked by 5546 edges (Figure 6A and Supplementary Dataset 2). Further information about the topological structure of CNC network is found in the Supplementary Dataset 3. Functional analysis of protein-coding genes in the network showed significant enrichment in various gene expression regulation processes, including rRNA processing, mRNA splicing, translational initiation, protein binding and RNA binding (Figure 6B, Supplementary Dataset 4, 5, 6). Taken together, the observations strongly suggested that the lncRNA genes related to the promoter D-DMRs might have influence on the corresponding target genes that with important functions during the human PED.

## DISCUSSION

DNA methylation is an important form of epigenetic modification and serves multiple critical functions, including repression of gene transcription, maintaining genomic integrity, establishing parent-specific imprinting patterns, and repression of transposable elements [18, 19]. Despite recent studies have profiled genome-scale maps of DNA methylation during human PED [20, 21], the specific dynamics of lncRNA genes were poorly described. In this article, we performed comprehensive analysis of the DNA methylation patterns of lncRNA genes in human PED.

Research on PED is important for both reproductive biology and regenerative medicine. Besides, understanding the nature of reprogramming and totipotency of early embryos will enlighten the research on and utilization of embryonic stem cells (ESCs) and induced pluripotent stem cells (iPSCs). However, PED is a special developmental stage, where a series of important distinctive developmental events happened, such as maternal-zygotic transition [41], ZGA [42], and segregation of inner cell mass and trophectoderm [43, 44]. These processes carry out the most dramatic genome-wide changes of DNA methylation in mammalian [20–22], and require accurate epigenetic regulation. Therefore, investigating of methylation features of lncRNA genes during PED is very important. In this study, we found that lncRNA genes shared many methylation characteristics with protein-coding genes during human PED, including methylation dynamics, TSS methylation distrbution, negative correlation between promoter and gene expression. Interestingly, lncRNA genes showed higher methylation levels in promoter than protein-coding genes during human PED. Therefore, it is reasonable to assume that methylation alterations at the promoters of lncRNA genes can change their expression levels, and in turn influences the expression of their downstream target genes by direct and indirect means.

Both sperm and oocytes contain gamete-specific methylation patterns [20–22]. Therefore the two haploid genomes arrive with diverse methylation signatures at the time of fertilization. Shortly after fertilization, the paternal genome undergoes quickly demethylation within the first several hours post-fertilization, and the maternal genome largely undergoes passive methylation [45]. However, whether there exist the differences in gamete-specific methylation patterns between lncRNA and protein-coding genes has not been studied. Here, we analyzed G-DMRs in lncRNA and protein-coding genes, and found that G-DMRs in lncRNA genes showed more paternal bias compared to protein-coding genes, especially in promoter regions. This indicated that lncRNA genes might play important roles in active DNA demethylation during PED. The paternal genome demethylation in lncRNA genes was similar to that in protein-coding genes in that the majority of methylation was rapidly lost, and the maternal genome in both two types of genes displayed slow demethylation which decrease over the course of PED.

The identification of DMRs across multiple samples is important in genomic function analysis [38]. Here, we performed D-DMRs analysis using QDMR [39], a bioinformatic tool for genome-wide quantitative comparisons of DNA methylation among multiple samples based on Shannon entropy. Then, we constructed a CNC network including the lncRNA and protein-coding genes related to the promoter D-DMRs based on transcriptome of human PED. The quality of the network and the accuracy of the function prediction is central to the network. In our CNC network, the strong enrichment in gene expression regulation process not only indicated the important function of these lncRNA genes with developmental specific methylation pattern during human PED, but also demonstrated the high-quality of our CNC network. The CNC network allows us to identify methylation regulated lncRNA and their affected targets whose expression is dynamically dependent on methylation states at lncRNA promoters. Such direct and indirect effects have been reported in transcription factors and miRNAs. In addition, based on the network topology measures, we could identify highly ranked hub genes. For example, we found many lncRNA genes linked to PWP1, which was nucleus located and function in histone modification [46]. We thus speculated that methylation alterations at the promoters of lncRNA genes can change their expression levels, and in turn influences the expression of their downstream target genes by direct and indirect means, including mRNA splicing, histone modification, transcription interferer, protein binding and RNA binding.

In summary, we provide the first methylation profiles of lncRNA genes during human PED. Several similarities between lncRNA and protein-coding genes were identified, including the methylation dynamics, TSS methylation distrbution, relationship between promoter and gene expression. The differences in promoter suggested the differential methylation regulation mechanisms between lncRNA and protein-coding genes. G-DMRs and D-DMRs analysis indicated that lncRNA genes contributed more to paternal methylation regulation than protein-coding genes. Our CNC network provide a large-scale network including epigenetically regulated lncRNA and their target genes for further biological research. Our results will help to understand the functions of lncRNA genes and their roles in human PED.

## MATERIALS AND METHODS

### Data resource

The reduced representation bisulphite sequencing (RRBS) dataset of human pre-implantation embryos (GSE49828) was downloaded from the Gene Expression Omnibus (GEO) of the National Center for Biotechnology Information. The dataset consists of 32 samples from each crucial stages of embryo development. The single-cell RNA-seq dataset of human pre-implantation embryos version is GSE36552, which includes 124 samples ranging from the oocyte to blastocyst stages.

The annotations of lncRNA and protein-coding genes were obtained from the hg19 Refseq and NONCODE2016, respectively. Promoter were defined as 1 kb up- and downstream of the transcription start sites (TSSs). The annotated regions, such as CGIs, exons and introns were downloaded from UCSC tables with hg19 track.

### RRBS and RNA-seq data analysis

For RRBS data, sequencing reads were aligned to the hg19 using Bismark tools (version 0.14.5) with default parameters. For RNA-seq data, reads were aligned to hg19 using TopHat version 2.0.9. FPKM (fragments per kilobase of exon per million fragments mapped) was computed with Cuffquant and Cuffnorm.

### Estimating methylation levels

The methylation level of each CpG site was estimated as the number of reads reporting a C, divided by the total number of reads reporting a C or T. Single CpG methylation levels were limited to those CpGs that had at least fivefold coverage. For 100-bp tiles, reads for all the CpGs that were covered more than fivefold within the tile were pooled and used to estimate the methylation level as described for single CpGs. The DNA methylation level of each sample is the average of the 100-bp tiles, while the DNA methylation level of each stage is the arithmetic average value of all biological replicates across each stage. The CpG density of every CpG site was calculated as the total numbers of all CpG dinucleotides located within 50 bp up- and downstream of that CpG site. The CpG density for a 100-bp tile is the average of the CpG density for all single CpGs usedto estimate methylation level in the tile.

### Identification of G-DMRs and D-DMRs

After quantifying the 100-bp tile DNA methylation levels using 100-bp-tile-based methylation calling algorithm, we systematically compared the DNA methylation levels of 100-bp tiles which were covered in both MII oocytes and sperm. We assigned these 100-bp tiles as G-DMRs only if the methylation level of these tiles is in one type of gametes greater than 75%, while in the other type ofgametes less than 25%, with a significant *P* < 0.05 given by multiple Student's t-test and a Benjamini-Hochberg false discovery rate (FDR) < 0.05.

We applied QDMR (version 1.0) to analyze D-DMRs with default parameters. Each region was assigned an entropy value by QDMR based on the methylation levels for all the samples. The regions whose entropy is less than the thrshold were identified as D-DMRs.

#### Construction of the coding–non-coding gene co-expression network

The single-cell RNA-seq dataset of human pre-implantation embryos were used to construct the coding-non-coding gene co-expression network, including following five steps: (1) we only keep the genes with maximal expression during PED more than 5 and expressional variance ranked in the top 75 percentile; (2) pearson correlation coefficient (Pcc) was computed using R ; (3) Pcc *P*-values for each gene pair was estimated through Fisher's asymptotic test implemented in the WGCNA library of R; (4) Keep only gens with the absolute value of Pcc > 0.8 and *P*-values < 0.05; (5) extract these gene pairs including the genes related to the promoter D-DMRs. The gene networks were visualized using Cytoscape 3.2.0.

#### Function enrichment analysis

The Database for Annotation, Visualization and Integrated Discovery (DAVID) was a frequently-used bioinformatics resources for GO functional annotation. First, we upload gene lists to DAVID. And then, after selecting identifier for thes genes (In this work, we select “ENSEMBL_GENE_ID”). Biological process, molecular fuction and cellular component terms was seleted as background gene sets respectively. Hypergeometric Exact test was used to measure gene-enrichment in background annotation terms.

## SUPPLEMENTARY MATERIALS FIGURES AND TABLES















## Abbreviations

PED: pre-implantation embryonic development
TSS: the transcription start sites of gene
DMRs: differentially methylated regions
G-DMRs: the DMRs between sperm and oocytes
D-DMRs: the DMRs across the human PED

## References

[1] Gendrel AV, Heard E (2014). Noncoding RNAs and epigenetic mechanisms during X-chromosome inactivation. Annu Rev Cell Dev Biol.

[2] Koerner MV, Pauler FM, Huang R, Barlow DP (2009). The function of non-coding RNAs in genomic imprinting. Development.

[3] Tsai MC, Manor O, Wan Y, Mosammaparast N, Wang JK, Lan F, Shi Y, Segal E, Chang HY (2010). Long noncoding RNA as modular scaffold of histone modification complexes. Science.

[4] Bertani S, Sauer S, Bolotin E, Sauer F (2011). The noncoding RNA Mistral activates Hoxa6 and Hoxa7 expression and stem cell differentiation by recruiting MLL1 to chromatin. Mol Cell.

[5] Ulitsky I, Shkumatava A, Jan CH, Sive H, Bartel DP (2011). Conserved function of lincRNAs in vertebrate embryonic development despite rapid sequence evolution. Cell.

[6] Hawkins PG, Morris KV (2010). Transcriptional regulation of Oct4 by a long non-coding RNA antisense to Oct4-pseudogene 5. Transcription.

[7] Guttman M, Donaghey J, Carey BW, Garber M, Grenier JK, Munson G, Young G, Lucas AB, Ach R, Bruhn L, Yang X, Amit I, Meissner A (2011). lincRNAs act in the circuitry controlling pluripotency and differentiation. Nature.

[8] Rosa A, Ballarino M (2016). Long noncoding RNA regulation of pluripotency. Stem Cells Int.

[9] Williams GT, Mourtada-Maarabouni M, Farzaneh F (2011). A critical role for non-coding RNA GAS5 in growth arrest and rapamycin inhibition in human T-lymphocytes. Biochem Soc Trans.

[10] Johnsson P, Ackley A, Vidarsdottir L, Lui WO, Corcoran M, Grander D, Morris KV (2013). A pseudogene long-noncoding-RNA network regulates PTEN transcription and translation in human cells. Nat Struct Mol Biol.

[11] Gomez JA, Wapinski OL, Yang YW, Bureau JF, Gopinath S, Monack DM, Chang HY, Brahic M, Kirkegaard K (2013). The NeST long ncRNA controls microbial susceptibility and epigenetic activation of the interferon-gamma locus. Cell.

[12] Wang P, Xue Y, Han Y, Lin L, Wu C, Xu S, Jiang Z, Xu J, Liu Q, Cao X (2014). The STAT3-binding long noncoding RNA lnc-DC controls human dendritic cell differentiation. Science.

[13] Wang K, Sun T, Li N, Wang Y, Wang JX, Zhou LY, Long B, Liu CY, Liu F, Li PF (2014). MDRL lncRNA regulates the processing of miR-484 primary transcript by targeting miR-361. PLoS Genet.

[14] Gibb EA, Brown CJ, Lam WL (2011). The functional role of long non-coding RNA in human carcinomas. Mol Cancer.

[15] Rapicavoli NA, Poth EM, Zhu H, Blackshaw S (2011). The long noncoding RNA Six3OS acts in trans to regulate retinal development by modulating Six3 activity. Neural Dev.

[16] Hamazaki N, Uesaka M, Nakashima K, Agata K, Imamura T (2015). Gene activation-associated long noncoding RNAs function in mouse preimplantation development. Development.

[17] Wang J, Li X, Wang L, Li J, Zhao Y, Bou G, Li Y, Jiao G, Shen X, Wei R, Liu S, Xie B, Lei L (2016). A novel long intergenic noncoding RNA indispensable for the cleavage of mouse two-cell embryos. EMBO Rep.

[18] Bird A (2002). DNA methylation patterns and epigenetic memory. Genes Dev.

[19] Fulka H, Mrazek M, Tepla O, Fulka J (2004). DNA methylation pattern in human zygotes and developing embryos. Reproduction.

[20] Guo H, Zhu P, Yan L, Li R, Hu B, Lian Y, Yan J, Ren X, Lin S, Li J, Jin X, Shi X, Liu P (2014). The DNA methylation landscape of human early embryos. Nature.

[21] Smith ZD, Chan MM, Humm KC, Karnik R, Mekhoubad S, Regev A, Eggan K, Meissner A (2014). DNA methylation dynamics of the human preimplantation embryo. Nature.

[22] Smith ZD, Chan MM, Mikkelsen TS, Gu H, Gnirke A, Regev A, Meissner A (2012). A unique regulatory phase of DNA methylation in the early mammalian embryo. Nature.

[23] Guo H, Zhu P, Wu X, Li X, Wen L, Tang F (2013). Single-cell methylome landscapes of mouse embryonic stem cells and early embryos analyzed using reduced representation bisulfite sequencing. Genome Res.

[24] Zhao J, Sun BK, Erwin JA, Song JJ, Lee JT (2008). Polycomb proteins targeted by a short repeat RNA to the mouse X chromosome. Science.

[25] Rinn JL, Kertesz M, Wang JK, Squazzo SL, Xu X, Brugmann SA, Goodnough LH, Helms JA, Farnham PJ, Segal E, Chang HY (2007). Functional demarcation of active and silent chromatin domains in human HOX loci by noncoding RNAs. Cell.

[26] Bond AM, Vangompel MJ, Sametsky EA, Clark MF, Savage JC, Disterhoft JF, Kohtz JD (2009). Balanced gene regulation by an embryonic brain ncRNA is critical for adult hippocampal GABA circuitry. Nat Neurosci.

[27] Wang X, Arai S, Song X, Reichart D, Du K, Pascual G, Tempst P, Rosenfeld MG, Glass CK, Kurokawa R (2008). Induced ncRNAs allosterically modify RNA-binding proteins in cis to inhibit transcription. Nature.

[28] Imamura T, Yamamoto S, Ohgane J, Hattori N, Tanaka S, Shiota K (2004). Non-coding RNA directed DNA demethylation of Sphk1 CpG island. Biochem Biophys Res Commun.

[29] Orom UA, Derrien T, Beringer M, Gumireddy K, Gardini A, Bussotti G, Lai F, Zytnicki M, Notredame C, Huang Q, Guigo R, Shiekhattar R (2010). Long noncoding RNAs with enhancer-like function in human cells. Cell.

[30] Beltran M, Puig I, Pena C, Garcia JM, Alvarez AB, Pena R, Bonilla F, de Herreros AG (2008). A natural antisense transcript regulates Zeb2/Sip1 gene expression during Snail1-induced epithelial-mesenchymal transition. Genes Dev.

[31] Durruthy-Durruthy J, Sebastiano V, Wossidlo M, Cepeda D, Cui J, Grow EJ, Davila J, Mall M, Wong WH, Wysocka J, Au KF, Reijo Pera RA (2016). The primate-specific noncoding RNA HPAT5 regulates pluripotency during human preimplantation development and nuclear reprogramming. Nat Genet.

[32] Li J, Gao Z, Wang X, Liu H, Zhang Y, Liu Z (2016). Identification and functional analysis of long intergenic noncoding RNA genes in porcine pre-implantation embryonic development. Sci Rep.

[33] Derrien T, Johnson R, Bussotti G, Tanzer A, Djebali S, Tilgner H, Guernec G, Martin D, Merkel A, Knowles DG, Lagarde J, Veeravalli L, Ruan X (2012). The GENCODE v7 catalog of human long noncoding RNAs: analysis of their gene structure, evolution, and expression. Genome Res.

[34] Sati S, Ghosh S, Jain V, Scaria V, Sengupta S (2012). Genome-wide analysis reveals distinct patterns of epigenetic features in long non-coding RNA loci. Nucleic Acids Res.

[35] Deaton AM, Bird A (2011). CpG islands and the regulation of transcription. Genes Dev.

[36] Yan L, Yang M, Guo H, Yang L, Wu J, Li R, Liu P, Lian Y, Zheng X, Yan J, Huang J, Li M, Wu X (2013). Single-cell RNA-Seq profiling of human preimplantation embryos and embryonic stem cells. Nat Struct Mol Biol.

[37] Edwards CA, Ferguson-Smith AC (2007). Mechanisms regulating imprinted genes in clusters. Curr Opin Cell Biol.

[38] Rakyan VK, Down TA, Thorne NP, Flicek P, Kulesha E, Graf S, Tomazou EM, Backdahl L, Johnson N, Herberth M, Howe KL, Jackson DK, Miretti MM (2008). An integrated resource for genome-wide identification and analysis of human tissue-specific differentially methylated regions (tDMRs). Genome Res.

[39] Zhang Y, Liu H, Lv J, Xiao X, Zhu J, Liu X, Su J, Li X, Wu Q, Wang F, Cui Y (2011). QDMR: a quantitative method for identification of differentially methylated regions by entropy. Nucleic Acids Res.

[40] Yagi S, Hirabayashi K, Sato S, Li W, Takahashi Y, Hirakawa T, Wu G, Hattori N, Ohgane J, Tanaka S, Liu XS, Shiota K (2008). DNA methylation profile of tissue-dependent and differentially methylated regions (T-DMRs) in mouse promoter regions demonstrating tissue-specific gene expression. Genome Res.

[41] Schier AF (2007). The maternal-zygotic transition: death and birth of RNAs. Science.

[42] Vassena R, Boue S, Gonzalez-Roca E, Aran B, Auer H, Veiga A, Izpisua Belmonte JC (2011). Waves of early transcriptional activation and pluripotency program initiation during human preimplantation development. Development.

[43] Bai Q, Assou S, Haouzi D, Ramirez JM, Monzo C, Becker F, Gerbal-Chaloin S, Hamamah S, De Vos J (2012). Dissecting the first transcriptional divergence during human embryonic development. Stem Cell Rev.

[44] Galan A, Montaner D, Poo ME, Valbuena D, Ruiz V, Aguilar C, Dopazo J, Simon C (2010). Functional genomics of 5- to 8-cell stage human embryos by blastomere single-cell cDNA analysis. PLoS One.

[45] Shen L, Inoue A, He J, Liu Y, Lu F, Zhang Y (2014). Tet3 and DNA replication mediate demethylation of both the maternal and paternal genomes in mouse zygotes. Cell Stem Cell.

[46] Honore B, Leffers H, Madsen P, Celis JE (1994). Cloning of a cDNA encoding a novel human nuclear phosphoprotein belonging to the WD-40 family. Gene.

