# Supplementary material for: DNA methylation signature of long noncoding RNA genes during human pre-implantation embryonic development
Source: Oncotarget. 2017 May 23;8(34):56829–38. doi: 10.18632/oncotarget.18072 (PMC5593605; doi:10.18632/oncotarget.18072)
Supplement: Supplementary file 1 [file oncotarget-08-56829-s001.pdf]

DNA methylation signature of long noncoding RNA genes during human pre-implantation embryonic development

SUPPLEMENTARY MATERIALS

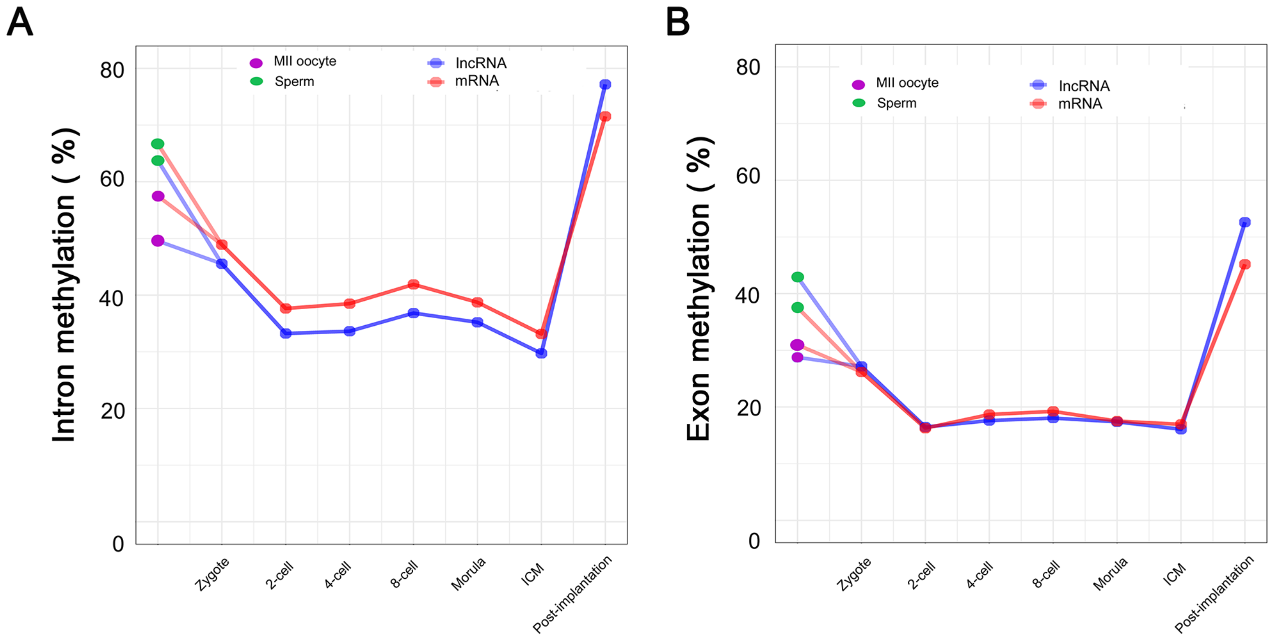

Supplementary Figure 1: Methylation level of lncRNA and protein-coding genes across each developmental stage. **(A)**, Intron. **(B)**, Exon. Blue, lncRNA. Red, protein-coding genes.

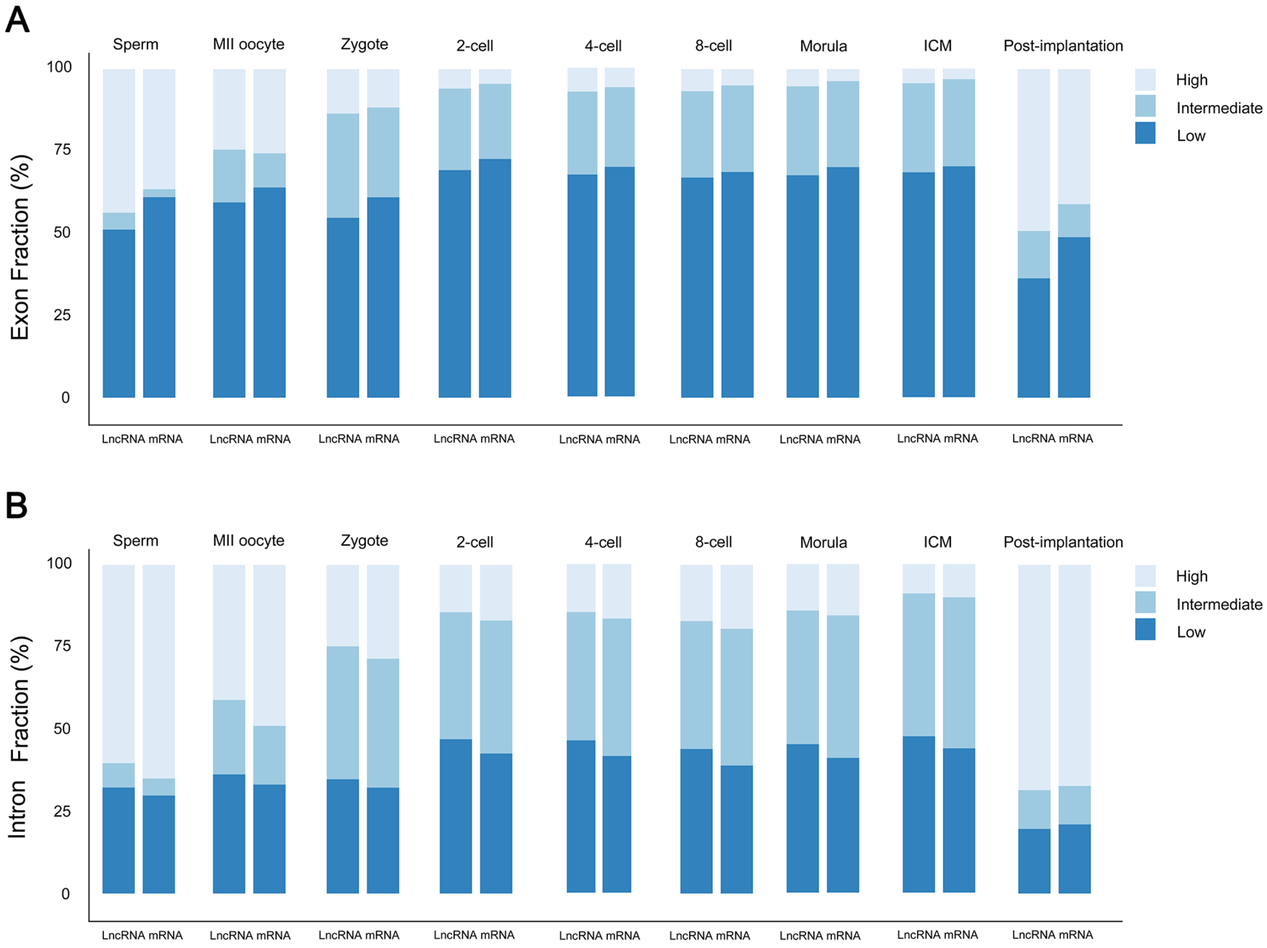

Supplementary Figure 2: Histogram of fractions of 100-bp tiles with three different methylation status across each developmental stages. (A), Intron. **(B)**, Exon.

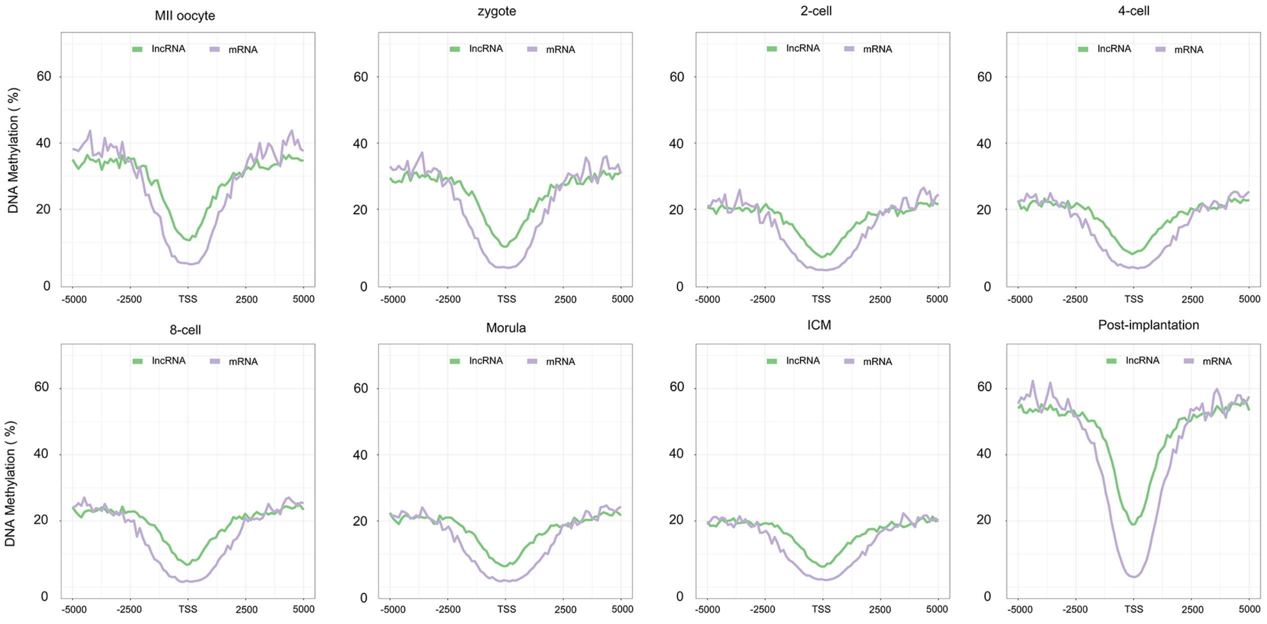

Supplementary Figure 3: DNA methylation patterns around the TSS of lncRNA genes.

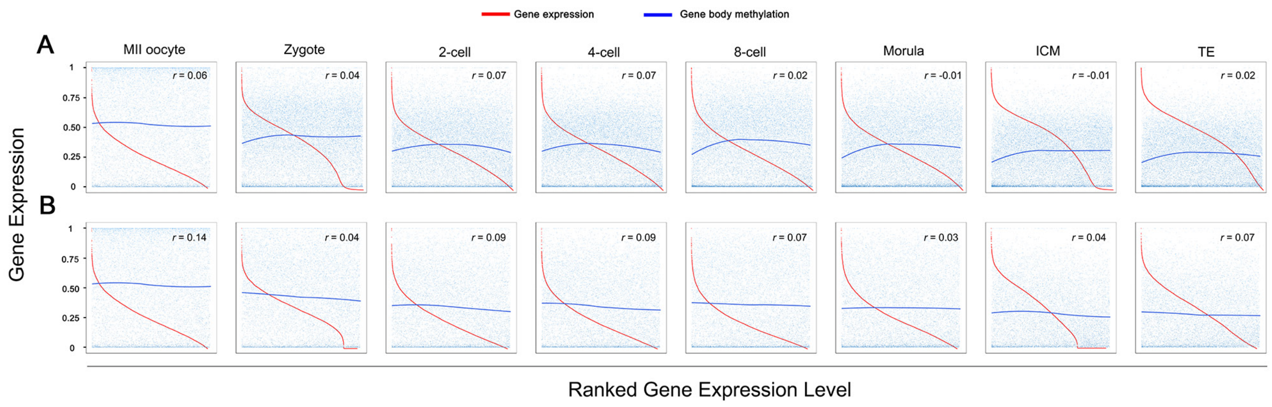

Supplementary Figure 4: Relationships between DNA methylation level of gene body and gene expression during human early embryonic development. (A), The scatter plot of DNA methylation levels of gene body regions and the relative expression levels of corresponding protein-coding genes. The $\log_2$ of the gene expression levels (fragments per kilobase per million) were calculated and are presented. The Pearson correlation coefficients (*r*) between DNA methylation levels of gene body regions and the scaled expression levels of the corresponding genes across every developmental stage are calculated and are included in the top right corner of each panel. The red and blue fitting curves in each display represent gene expression levels and DNA methylation levels in gene body regions, respectively. The horizontal axis from left to right below each box represents the expression levels from high to low of protein-coding genes, respectively. **(B)**, The same in (A) of lncRNA genes.

Supplementary Dataset 1: The detail results of QDMR.

See Supplementary File 1

Supplementary Dataset 2: The pearson correlation coefficient matrix of gene pairs in CNC network.

See Supplementary File 2

Supplementary Dataset 3: The detail results of topological structure of CNC network.

See Supplementary File 3

Supplementary Dataset 4: GO analysis of protein-coding genes in CNC network for biology process term.

See Supplementary File 4

Supplementary Dataset 5: GO analysis of protein-coding genes in CNC network for molecule function term.

See Supplementary File 5

Supplementary Dataset 6: GO analysis of protein-coding genes in CNC network for cellular component term.

See Supplementary File 6
